# A global database of water vapor isotopes measured with high temporal resolution infrared laser spectroscopy

**DOI:** 10.1038/sdata.2018.302

**Published:** 2019-01-22

**Authors:** Zhongwang Wei, Xuhui Lee, Franziska Aemisegger, Marion Benetti, Max Berkelhammer, Mathieu Casado, Kelly Caylor, Emanuel Christner, Christoph Dyroff, Omaira García, Yenny González, Timothy Griffis, Naoyuki Kurita, Jie Liang, Mao-Chang Liang, Guanghui Lin, David Noone, Konstantin Gribanov, Niels C. Munksgaard, Matthias Schneider, François Ritter, Hans Christian Steen-Larsen, Christine Vallet-Coulomb, Xuefa Wen, Jonathon S. Wright, Wei Xiao, Kei Yoshimura

**Affiliations:** 1 School of Forestry and Environmental Studies, Yale University, New Haven, CT, USA; 2 River and Environmental Engineering Lab, Department of Civil Engineering, The University of Tokyo, Tokyo, Japan; 3 Yale-NUIST Center on Atmospheric Environment, Nanjing University of Information Science & Technology, Nanjing, Jiangsu, China; 4 Institute for Atmospheric and Climate Science, ETH Zurich, Zurich, Switzerland; 5 Institute of Earth Sciences, University of Iceland, Reykjavik, Iceland; 6 Department of Earth and Environmental Sciences at University of Illinois at Chicago, Chicago, Illinois, USA; 7 Laboratoire des Sciences du Climat et de l’Environnement, LSCE/IPSL, CEA-CNRS-UVSQ, Université Paris-Saclay, Gif-sur-Yvette, France; 8 Department of Geography, Bren School of Environmental Science & Management, University of California - Santa Barbara, Santa Barbara, CA, USA; 9 Institute of Meteorology and Climate Research (IMK-TRO), Karlsruhe Institute of Technology, Karlsruhe, Germany; 10 Aerodyne Research Inc., Billerica, MA, USA; 11 Izaña Atmospheric Research Centre (IARC), Agencia Estatal de Meteorología (AEMET), Santa Cruz de Tenerife, Canary Islands, Spain; 12 Department of Earth Atmospheric and Planetary Sciences, Massachusetts Institute of Technology, Cambridge, MA, USA; 13 Department of Soil, Water, and Climate, University of Minnesota, Twin Cities, Saint Paul, MN, USA; 14 Graduate School of Environmental Studies, Nagoya University, Nagoya, Aichi, Japan; 15 Department of Earth System Science, Tsinghua University, Beijing, China; 16 Research Center for Environmental Changes, Academia Sinica, Taiwan; 17 College of Earth, Ocean and Atmospheric Sciences, Oregon State University, Corvallis, OR, USA; 18 Ural Federal University, Ekaterinburg, Russia; 19 Research Institute for the Environment and Livelihoods, Charles Darwin University, Darwin, Northern Territory, Australia; 20 Institute of Meteorology and Climate Research (IMK-ASF), Karlsruhe Institute of Technology, Karlsruhe, Germany; 21 Geophysical Institute, University of Bergen and Bjerknes Centre for Climate Research, Bergen, Norway; 22 Aix Marseille Université, CNRS, IRD, CDF, CEREGE UM 34, 13545 Aix en Provence, France; 23 Key Laboratory of Ecosystem Network Observation and Modeling, Institute of Geographic Sciences and Natural Resources Research, Chinese Academy of Sciences, Beijing, China; 24 Institute of Industrial Science, The University of Tokyo, Komaba, Tokyo, Japan

**Keywords:** Hydrology, Atmospheric chemistry

## Abstract

The isotopic composition of water vapour provides integrated perspectives on the hydrological histories of air masses and has been widely used for tracing physical processes in hydrological and climatic studies. Over the last two decades, the infrared laser spectroscopy technique has been used to measure the isotopic composition of water vapour near the Earth’s surface. Here, we have assembled a global database of high temporal resolution stable water vapour isotope ratios (δ^18^O and δD) observed using this measurement technique. As of March 2018, the database includes data collected at 35 sites in 15 Köppen climate zones from the years 2004 to 2017. The key variables in each dataset are hourly values of δ^18^O and δD in atmospheric water vapour. To support interpretation of the isotopologue data, synchronized time series of standard meteorological variables from in situ observations and ERA5 reanalyses are also provided. This database is intended to serve as a centralized platform allowing researchers to share their vapour isotope datasets, thus facilitating investigations that transcend disciplinary and geographic boundaries.

## Background & Summary

Water vapour in the atmospheric boundary layer, the lowest layer of the Earth’s atmosphere and about 1 km in thickness, is a key component of the hydrological cycle and the climate system. It is estimated that this reservoir contains the majority of the atmospheric vapour, contributing to about 2/3 of the Earth’s natural greenhouse effect^1–3^. Water vapour mixing ratios are expected to rise in the future warmer climate, leading to more extreme weather events^4^. Quantification and elucidation of processes underlying the variations in atmospheric water vapour remain one of the grand challenges in water cycle science^5^.

There are four primary isotopologues of water: H_2_^16^O (about 99.731%), HD^16^O (about 0.003789%), H_2_^17^O (0.037%) and H_2_^18^O (about 0.2%)^6^. Isotopic compositions indicate the relative abundance of a heavier isotope (HD^16^O or H_2_^18^O) to the lighter H_2_^16^O isotope. Kinetic and equilibrium fractionation processes occur during transitions between the vapour phase and the condensed phases (liquid or solid), such that the vapour phase is generally more depleted in the heavier isotopes than the condensed phases. The degree of fractionation is strongly influenced by the atmospheric conditions (temperature, humidity, and level of turbulence) during the phase transitions. The isotopic compositions of water vapour can therefore provide an integrated perspective on the hydrological history of air airmasses and has been widely used for tracing physical processes in hydrological and climatic studies^7^. Water vapour isotopic measurements provide additional constraints on state variables of the atmosphere simulated by general circulation models^8–10^. By tagging atmospheric moisture with the isotopic tracers, it becomes possible to trace the origin of precipitation water with isotope-enabled Lagrangian trajectory models^11,12^. By incorporating information on vapour isotopes, an atmospheric data assimilation system yields more accurate simulations for meteorological fields than if such information is left out^13^.

Currently, two databases on stable water isotopes are publicly available. The Global Network of Isotopes in Precipitation (GNIP), administrated by the International Atomic Agency, has been archiving precipitation isotope data for several decades^14^. The Global Network of Isotopes in Rivers (GNIR) is another public data depository developed recently for archiving isotopic compositions of river water^15^. Both databases are for isotopes in the condensed phases of water. To our knowledge, our database is the first to archive vapour isotope measurements for public use without restriction.

In this paper, we describe a Stable Water Vapour Isotope Database (SWVID) that archives high-frequency observations of water vapour isotopes collected using isotopologue-resolved infrared laser spectroscopy. Before this technology became available, measurements of vapour isotopic compositions required collection of water vapour using cryogenic trapping and subsequent analysis by mass spectrometry, a labor-intensive operation that does not permit high-frequency measurements. Thanks to the development of research-grade and commercial water vapour isotope spectroscopic analysers, it is now possible to measure isotopic compositions of water vapour continuously, in-situ, and at high temporal frequencies (seconds to hours). Such measurements have improved our understanding of the mechanisms underlying hydrological processes, such as diagnosing moisture paths, partitioning evapotranspiration, and interpreting convective mixing^16^. The SWVID is intended to serve as a platform similar to GNIP and GNIR, allowing researchers to share their vapour isotope datasets at a centralized depository and thus facilitating investigations that transcend disciplinary and geographic boundaries. We expect that data in this archive will be useful for 1) validation of isotope-enabled atmospheric models, 2) calibration of vapour isotope observations from satellites, 3) investigation of isotopic fractionations under varied surface conditions, and 4) improvement of atmospheric data assimilation.

**Figure 1:**
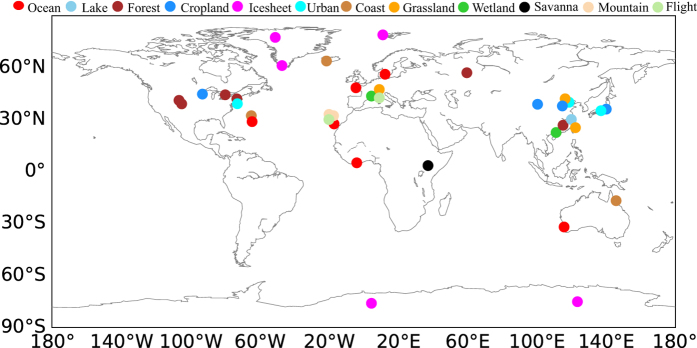
Map of measurement sites as of March 1, 2018. Initial point of ship cruises is marked as Ocean.

**Figure 2:**
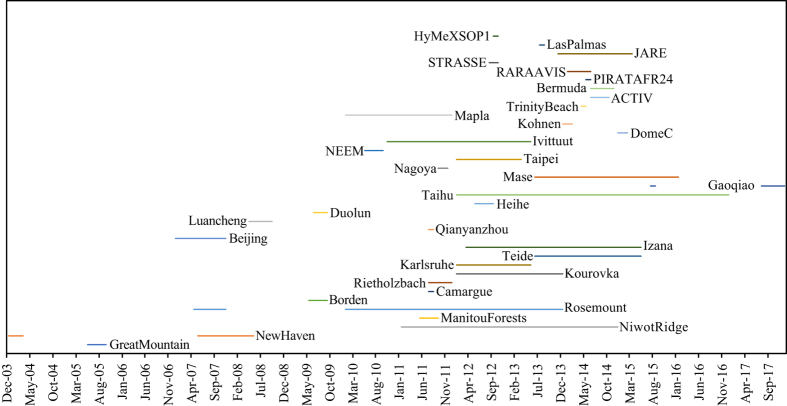
The measurement period for each site.

As of March 2018, the datasets in this database (Data Citation 1) have been collected in 15 Köppen climate zones (Fig. 1 and Table 1, also see http://vapor-isotope.yale.edu/). The key variables in each dataset are ^18^O and D isotopic compositions of atmospheric water vapour. To support interpretation of the isotopic data, synchronized time series of standard meteorological variables (atmospheric pressure, precipitation amount, net radiation, relative humidity, air temperature, and wind speed and direction) are also provided. The measurement period for each site is shown in Fig. 2.

## Methods

We have compiled in-situ water vapour isotope measurements and the corresponding climatic data from 35 sites (Fig. 1 and Table 1). Measurement durations range from 1 week to 3 years between 2004 and 2017. Below we describe biogeographic characteristics and measurement details of each site, organized by geographic region. The instruments used are described in Tables 2 and 3. Not all the sites had simultaneous measurements of standard meteorological variables; at these sites, the data gaps were filled with -9999. In addition, ERA5 hourly data (Data Citation 2) was also provided as a reference or can be used for gap filling. ERA5 data span the time period from 1950 to present with a 0.25° × 0.25° grid size. ERA5 is a long-term global reanalysis dataset that assimilates vast amounts of historical observations in the climate system^17^. All isotope compositions are expressed in delta notation in reference to the VSMOW (Vienna Standard Mean Ocean Water) standard.

### North America

Great Mountain (41.98° N, 73.23° W, elevation 460 m above mean sea level) is a forest site^18^ located in Connecticut, United States. The forest is dominated by red maple, eastern white pine, beech and hemlock. Mean tree height varies between 16 and 22 m. The average annual temperature is 7.0° C, with the average total annual precipitation of 133 cm. The water vapour isotope experiment was conducted from May 2005 to September 2005, using a tunable diode laser (TDL) trace gas analyzer (TGA100A, Campbell Scientific Inc., Logan, Utah, USA). The analyzer measured the isotopic composition at 10 Hz and the data were averaged to hourly intervals for archiving and analysis. The measurement height was 30.7 m above the ground. Standard meteorological variables were measured simultaneously at the forest.

New Haven (41.30° N, 72.92° W, elevation 20 m above mean sea level) is an urban site^19^ located on the coast of the Atlantic Ocean in Connecticut, USA. Water vapour isotope experiments were conducted from December 2003 to December 2004 and from March 2007 to May 2008. The isotopic composition of atmospheric vapour was measured with the same TDL analyzer as Great Mountain. The air was drawn from 21.7 m above the ground. The average annual temperature is 10.5° C. The average annual precipitation is 120 cm. Standard meteorological variables were measured at the Tweed Airport 10 km away from the isotopic measurement site.

Rosemount (44.70° N, 93.08° W, elevation 294 m above the mean sea level) is a farmland site^20–22^, located at the Rosemount Research and Outreach Centre, the University of Minnesota, near the Minneapolis – St Paul, Minnesota, USA. The crop species are corn and soybean, which are typical of the US Corn Belt. The annual mean air temperature is 7.6 °C and precipitation amount is 92 cm. The site includes two isotopic measurement datasets. One dataset was obtained from April to October 2007 at the G21 site, which is part of the AmeriFlux network, and the other was obtained at tall tower site from January 2010 to April 2014. The instrument at G21 was a TDL analyzer (model TGA100A, Campbell Scientific Inc.) and that at the tall tower site was also a TDL analyzer (model TGA200, Campbell Scientific Inc.). The sample inlets were located at a height of 185 m and 3 m above the ground surface at the tall tower and the G21 site, respectively. The instrumental temporal resolution is 2 Hz. The averaging interval is 1 h.

Borden (44.32 °N, 79.93 °W, 240 m above mean sea level) is an Environment Canada research station located in a forest site^23^ in Ontario, Canada. The forest is composed of red maple, eastern white pine, large-tooth aspen and white ash. The mean tree height is 21 m. Annual mean air temperature is 6.4 °C and annual mean precipitation is 86 cm. The measurement was made with a TDL analyzer (model TGA100A) with the intake height of 37 m above the ground. The sampling frequency is 2 Hz and the averaging interval is 1 h. The experiment was carried out from May to August 2009.

Niwot Ridge (40.03° N, 105.55° W, 3050 m above mean sea level)^24^ is a forest site located in Colorado, Unite States and operated as part of the AmeriFlux network. This is a subalpine mixed coniferous forest (*Abies Lasiocarpa, Picea Engelmanni and Pinus Contorta*) with a mean tree height of 11 m. A cavity-enhanced near infrared laser (CIL) analyzer (model L2120i, Picarro Inc., Sunnyvale, California, USA) was used to measure water vapour isotopes at 1 Hz. The averaging interval is 1 h. The height of the sampling inlet is 20 m above the ground.

Manitou Experimental Forest (39.08° N, 105.07° W, 2503 m above mean sea level)^24,25^ is located in Colorado, USA. The site is dominated by a mature open-canopy ponderosa pine forest with an average height of 18.5 m. The experiment was conducted from May to September 2011 using a CIL isotopic analyzer (model L2120i, Picarro Inc.) with the inlet positioned at a height of 27.1 m above the ground. The sampling frequency is 0.6 Hz, and the averaging interval is 1 h.

### Europe

Currently six datasets in Europe are available in our database, including Camargue in France, Rietholzbach in Switzerland, Kourovka in Russia, Karlsruhe in Germany, and Teide and Izana in Spain.

At the Camargue site (43.60° N, 4.47° E; 10 m above mean sea level)^26^, continuous measurement of the isotopic compositions of atmospheric water vapour was performed between July and August 2011. The site is 170 m away from a lagoon and 12 km from the Mediterranean Sea. This area is controlled by the Mediterranean climate. The annual mean temperature is 14.0° C. The annual precipitation is 70 cm. The Camargue site is situated in a vast wetland area consisting of variable saltpans, lakes, lagoons, saline, freshwater marshes and temporary wetlands. The site is flooded during the wet season (September – March) while is dry in other months. The isotopic analyzer type is CIL (model L1102i, Picarro). The sampling frequency is 0.2 Hz, the averaging time is 1 h and the air intake height is 1.75 m above the ground.

The Kourovka site (57.04° N, 59.55° E; 300 m above mean sea level)^27^ is located in the Kourovka Astronomical Observatory near the western boundary of western Siberia, Russia. This site is dominated by pristine peatland with dense pine forests. The mean tree height is about 15 m. Local climate is continental. The annual mean temperature is −1.0 °C and the annual mean precipitation is 46 cm. The water vapour isotopic measurement was performed from September 2012 to August 2013 with a CIL analyzer (Model L2130i, Picarro). The sampling frequency is 2 Hz, the averaging interval is 1 h, and the inlet height is 8 m above the ground.

The Rietholzbach site is situated in the northeastern Swiss Prealps (47.37° N, 8.99° E, 755 m above the mean sea level). The catchment is sparsely populated and primarily used as pastureland, while about 25% of the area is forested^28^. The Rietholzbach site has a temperate humid climate, with an annual mean precipitation is 146 cm and the mean temperature is 7.1°C. The water vapour isotope measurement was performed from August to December 2011 with a CIL analyzer (model Picarro L1115i, Picarro). The sampling frequency is 0.2 Hz, the averaging interval is 1 h, and the intake height is at 1.5 m above the ground.

The Karlsruhe site is located 12 km north of Karlsruhe in southwestern Germany (49.10° N, 8.44° E; 110.4 m above mean sea level)^29^. The measurement was conducted in an urban area near the KIT campus. The site has an oceanic climate, with an annual mean precipitation is 79 cm and the mean temperature is 11 °C. The analyzer type is CIL (model L2120i, Picarro). The sampling frequency is 0.6 Hz, the averaging interval is 1 h, and the inlet height is 28 m above the ground level. The measurement took place between September 2012 to August 2013.

Izana and Teide are two mountain sites on Canary Islands, Spain^30^. The area where these two sites are situated has a Mediterranean climate. The vegetation in the surrounding area consists of sparse brooms. The Izana Observatory is a mountain-top station located at 2367 m above the mean sea level (28.3° N, 16.5° W), and the Teide Observatory is located at a volcanic cone at 3550 m above mean sea level (28.3° N, 16.5° W). During the measurement period, air temperature varied between −6° C and 15° C at Izana and between 6° and 11 °C at Teide. The annual mean precipitation, measured at a weather station (52 m above mean sea level) on Canary Islands, is 23 cm^31^. The analyzer type at both stations is CIL (model L2120i, Picarro). The sampling frequency is 0.6 Hz and the averaging interval is 1 h. The measurement started at Izana in March 2012 and that at Teide in July 2013 and they are still ongoing. The most recent data are available upon request to the current station PI, O. E. Garcia. At Izana, the sampling inlet is at a height of 30 m above the ground. At Teide, the inlet height is 6 m above the ground.

### Asia

Currently, we have eight sites in Asia, including eight sites in China (Beijing, Qianyanzhou, Luancheng, Duolun, Heihe, Taihu, and Gaoqiao), two sites in Japan (Mase and Nagoya) and one site in Taipei.

At the Beijing site (40.00° N, 116.23° E, 45 m above the mean sea level), the measurement was made from December 2006 to December 2007 in an urban neighourhood^32,33^. The annual mean air temperature is 12 °C. The annual mean precipitation is 58 cm. A TDL trace gas analyzer (model TGA100A, Campbell Scientific) was used in this site. The sampling frequency is 10 Hz, the averaging interval is 1 h, and the inlet height is at 10 m above the ground.

Qianyanzhou (26.75° N, 115.05° E, 102 m above the mean sea level) is a forest site operated as part of the Chinese Ecosystem Research Network (CERN)^34^ and the ChinaFLUX eddy flux network, in southeast China. The dominant vegetation types are slash pine and cypress, with a mean height of 14.3 m. The annual mean temperature is 17.1 °C. The annual mean precipitation is 138 cm. The isotopic measurements were conducted from December 2006 to December 2007, using a CIL analyzer (model DLT100, Los Gatos Research, Mountain View, California). The sampling frequency is 2 Hz, the averaging interval is 1 h, and the air intake height is 27 m above the ground.

The Luancheng site (37.83° N, 114.67° E, elevation 50 m above mean sea lavel)^35–37^ is a winter wheat and summer maize double-cropping system in north China. This site is also part of the CERN. Wheat was planted in November 2007 and harvested in mid-June 2008, with a maximum height of 0.75 m. Corn was planted at the beginning of June 2008 and was harvested in mid–September 2008. The annual mean temperature is 12.2 °C. The annual mean annual precipitation is 48 cm. The isotopic experiment was conducted between April and September 2008, using a TDL analyzer (model TGA100A, Campbell Scientific). The sampling frequency is 10 Hz, the averaging interval is 1 h, and the inlet height is 1.6 m to 4.2 m above the ground depending crop height.

The Duolun experiment was conducted in a steppe ecosystem in northwest China at the Duolun Restoration Ecology Research Station (42.05° N, 116.28° E, elevation 1,324 m above mean sea level)^36^, Institute of Botany, Chinese Academy of Sciences. The dominant vegetation species are *Stipa krylovii*, *Agropyron cristatum*, and *Artemisia frigida*. The mean air temperature is 2.1 °C. The annual mean precipitation is 38 cm. The isotopic measurement was conducted from June to September 2009 using a TDL analyzer (model TGA100A, Campbell Scientific). The sampling frequency is 10 Hz, the averaging interval is 1 h, and the inlet height is 1.8 m above the ground.

The Heihe site, located in in the middle reaches of the Heihe River watershed (38.85° N 100.37° E, 1,550 m above mean sea level) in northwest China, is part of the Heihe Watershed Allied Telemetry Experimental Research (HiWATER) program^38^. The experiment was conducted in an arid oasis spring maize, with an area of 5.5 km × 5.5 km. The annual mean temperature is 7.4° C and the mean annual precipitation is 13 cm. The isotopic experiment was conducted from May to September in 2012, using a CIL analyzer (model L2120i, Picarro). The sampling frequency is 0.6 Hz, and the averaging interval is 1 h. The intake height is 1.5 m to 3.6 m above the ground, depending on crop height.

The Taihu site (31.4° N, 120.22° E) is part of the Taihu Eddy Flux Network. Lake Taihu is a large (area 2400 km^2^) and shallow (mean depth 1.9 m) freshwater lake in the Yangtze River Delta, China^39^. The annual mean air temperature is 16.2°C and annual mean precipitation is 112 cm. The measurement took place over the water, at a location about 250 m from the north shore (31.42° N, 120.21° E, with a water depth of 1.8 m). The analyzer type is DLT (model DLT100, Los Gatos Research, Mountain View, California). The air inlet height is 3.5 m above the water surface. The sampling frequency is 2 Hz, and the averaging interval is 1 h. The measurement started in August 2014 and ended in December 2016.

At the Gaoqiao site (21.57°N, 109.76°E, 1 m above mean sea level), the isotopic measurement was performed in July 2015. The experimental site is located in the Zhanjiang Mangrove National Nature Reserve, close to Yingluo Bay, near Beihai city. This area is influenced by tropical monsoon climate and marine climate, with distinct wet and dry seasons. The total annual rainfall is about 160 cm. The annual mean temperature is 23.0 °C. The Gaoqiao site is a vast wetland consisting of six mangrove species with a mean canopy height of 1.5 m. The analyzer type is CIL (Picarro L2130-i). The sampling frequency is 0.2 Hz, and the averaging interval is 1 h. The analyzer air intake is located at a height of 4 m above the plant canopy.

The Mase site (36.03° N, 140.01° E, 12 m above the mean sea level), which is part of the AsiaFlux eddy flux network, is located in a rice paddy field in Tsukuba, Japan^40,41^. The field is sparsely covered by weeds in the spring (March-April), and by rice plants from May until harvesting at the end of August. The maximum canopy height is 1.1 m. The mean annual air temperature of 13.8 °C and the mean annual precipitation is 120 cm. The isotopic measurement was made with a CIL analyzer (model L2120i, Picarro) with an inlet height of 2 m above the ground. The sampling frequency is 0.6 Hz, and the sampling interval is 1 h.

Nagoya is an urban site located in an urban neighborhood of the City of Nagoya (35.15° N, 136.97° E, 50 m above the mean sea level) on the Pacific side of central Japan. The mean annual temperature is 16.0 °C. The mean annual precipitation is 150 cm. The isotopic measurement was conducted from September to November 2011. The inlet height is 15 m above the ground. The analyzer type is DLT (model DLT-100)^42,43^, with a sampling frequency of 2 Hz.

Water vapour measurement at the Taipei site was carried out with the analyzer’s inlet above a cabin top (inlet height about 3 m above the ground) in the grassland (25.01N, 121.54E), managed by the Department of Atmospheric Science, National University of Taiwan (NUT), from 11th January to 14th August 2012) and with the inlet at the 7th floor (inlet height 30 m above the ground) of the Department of Geography building on the NUT campus, from 15th August 2012 to 31st March 2013^44^. The distance between the two sites is about 200 m. These sites are located in the City of Taipei (25.18° N, 121.67° E, 10 m above the mean sea level) on the western Pacific side of northern Taiwan. The annual mean temperature is 23.0 °C. The mean annual precipitation is 240.5 cm. The analyzer type is CIL (model L2120i, Picarro). The sampling frequency is 0.6 Hz. Meteorological data are obtained from a nearby meteorology station operated by the Center for Weather Bureau, Taiwan (site number: 466920).

### Africa

The Mpala Research Center located in Kenya is operated by Princeton University^45,46^. The isotopic measurement started in February 2010 at an eddy covariance tower in the Center (0.49° N, 36.87° E, 1619 m above the mean sea level). This site is located in a semiarid mixed savanna ecosystem, which receives an annual mean rainfall of about 50 cm. The annual mean temperature is 20.3° C. The vegetation surrounding the tower has an average height of approximately 4 m and is a sparse mixture of mainly Acacia woody species and C4 grasses. The measurement was made with a CIL analyzer (model DLT-100, LGR) at a sampling frequency of 1 Hz. The analyzer’s intake height is 22.5 m above the ground.

### Oceania

The isotope measurement was made at Trinity Beach, Australia (16.79° S, 145.70° E, 20 m above the mean sea level) from April 10 to 13, 2014, using a CIL analyzer (model L2130i, Picarro) at a sampling frequency of about 1 Hz^47^. The intake height is 3 m above the ground. The mean annual maximum (minimum) temperature is 29.0 (20.8) °C. The mean annual rainfall is about 199 cm. The measurement was conducted over an urban area. The experiment experienced the passage of Cyclone Ita. The averaging interval is 25 seconds.

### Greenland

Atmospheric isotopic monitoring was carried out as part of the international deep drilling program at the NEEM site^48^ in northwest Greenland (77.45° N, 51.05° W, 2484 m above the mean sea level) during the summer field campaigns of 2010 (24 May to 3 August), 2011 (5 July to 3 August), and 2012 (21 May to 3 August). The annual mean precipitation is about 20 cm. The annual mean air temperature is about −29 °C, with summer mean temperature of –8.3° C. Water vapour isotope observations were conducted at a 2 Hz with an LGR DLT100 analyzer and a 0.6 Hz Picarro L2120i analyzer (2010 season) and a Picarro L2120i analyzer (2011 and 2012 seasons). The intake height is 1 m and 13 m (2010 season) and 3 m (2011 and 2012 season) above the snow surface. The averaging interval is 24 h.

Ivittuut, southern Greenland (61.21° N, 48.17° W, 30 m above the mean sea level) is a coastal site 100 m away from the Arsuk Fjord, at approximately 5 km east of the open ocean and 10 km west of the Greenland Ice Sheet. Arctic vegetation, mainly bushes and grasslands, is present in at the site. The annual mean temperature is 2 °C. The annual mean precipitation is 113 cm. The measurement started in September 2011 and ended in September 2012. The instrument type is CIL (model L2120i, Picarro). Its inlet height is 5 m above the ground. The sampling frequency is 0.6 Hz, and the averaging interval is 24 h.

### Antarctica

The Concordia station is located near the top of Dome C (75.10° S, 123.38° W, 3233 above the mean sea level), about 950 km from the coast^49^. While the local mean temperature is –54.3 °C, it was –32.4 °C during the campaign of 2014/2015, reaching a maximum value of –24.5 °C. Ice core data suggest an average annual snow accumulation of 2.7 ± 0.7 g cm^–2^ yr^–1^. The isotopic measurement was conducted from December 2014 to January 2015 with a CIL analyzer (model L2130i, Picarro) at a sampling frequency of 2 Hz. The averaging period is 1 h. The inlet height is 2 m above the snow surface.

The Kohnen station (75.00° S, 0.07° E) is a summer station located on the Antarctic Plateau, 2892 m above sea level and 550 km from the coast. The surface slope on this site is 1.3 ± 0.3 m km^−1^ and typical Katabatic winds form around 3 h and vanish around 15 h local time. The mean temperature for the 2014 campaign was similar to the average from 1998 to 2013 (−25° C). Water vapour isotope measurements were performed using a Los Gatos Research (LGR) Inc. analyzer (type DTL-100) from 17^th^ December 2013 to 21^th^ January 2014. The sampling inlet height is 3 m, the sampling frequency is 2 Hz and the average period is 11 min.

### Flight and Ship cruises

The vapour isotopic measurement was conducted on the icebreaker Shirase along the Japanese Antarctic Research Expedition (JARE) cruise track during two Australian summers in 2013/2014 (JARE55) and 2014/2015 (JARE56)^50^. On each cruise, the ship left Fremantle in Western Australia in late November, sailed to the Syowa station (69.00° S, 39.58° E) on East Ongul Island, Antarctica in late December, and stayed in the Lutzow-Holm Bay near Syowa to early February. The ship then sailed back to Australia, arriving at Sydney in Eastern Australia or Fremantle by the middle of March. The measurement was made on the top deck of the ship with a LGR TDL100 analyzer. The inlet height is 30 m above the sea surface. The sampling frequency is 2 Hz, and the averaging interval is 1 h. Hourly meteorological variables were measured at the height of 30 m above the sea surface.

Isotopic measurements were made in the Atlantic marine boundary layer on five research cruises, including STRASSE, PIRATAFR24, RARA, ACTIV and BERMUDA, between 2012 and 2015. These measurements were made with laser spectroscopy (Picarro model L2130i, L2130i, L2120i, L1102i and L2120i for STRASSE, PIRATA FR 24, RARA, ACTIV and BERMUDA, respectively). The analyzer’s inlet height varies between 10 and 20 m above the sea level. These cruises took place in the Atlantic Ocean between 4° S to 63° N. The average period is 15 min. More detailed information about these five cruises is given by Benetti *et al*.^51^ and the data (isotopic and meteorological measurements) are also available at Data Citation 3.

Vertical profiles of water vapor isotopes were measured during the MUSICA project over the Atlantic Ocean near the Canary Islands, Spain. Seven research flights were conducted from the Las Palmas Airport (27.93° N, −15.38° E) on 21, 22, 24, 25, 30, 31 July and 01, August 2013^52^. Measurements of water vapor mixing ratio and its deuterium isotope ratio δD were made with a custom-designed tunable diode laser spectrometer ISOWAT II at altitudes between 150 m and about 7000 m above sea level. The sampling frequency is 1 Hz. A custom designed working reference generator was used to perform in-flight measurements of a certified water working reference. Extensive pre- and post-flight calibrations were conducted on the ground with a custom designed calibration unit. The in-situ data measured during MUSICA are freely available at: http://share.lsdf.kit.edu/imk/asf/musica/INSITU/.

Vertical profile measurements were made during the HyMeX SOP1 campaign in the western Mediterranean^53^, on board of a research aircraft in September to November 2012 (Data Citation 4). The flight altitude varies from 0 to 4500 m above the ground surface. The instrument is Picarro L2130i analyzer customized with a triple-laser setup to allow fast measurement. The data acquisition rate is 1 Hz.

## Data Records

The isotopic and meteorological data are archived at Data Citation 1 and an interactive map of these data are available at http://vapor-isotope.yale.edu/.

### Variable Table

The isotopic and meteorological data are recorded in comma separated ascii- format in csv files, and the meta information is stored as PDF files. The meta file contains information about time stamps, data format, time zone convention, missing data flags, variable definitions, reference papers and site contact.

Variable names of point measurement in these csv files and measurement details are described in Table 3, using the Mase site as an example. Data variables are stored at hourly resolutions marked by the start and the end time stamps using the format YYYYMMDDHHmm (except for Trinity Beach site, which is recond in YYYYMMDDHHmmss), where YYYY, MM, DD, HH, mm and ss denote year, month, date, hour, minute and second, respectively. Time is reported as UTC standard time. Missing data is replaced with -9999. Data columns from stationary sites are in the following order: start time, end time, water vapor mixing ratio, δ^18^O, standard deviation of δ^18^O, δD, standard deviation of δD, air temperature, relative humidity, air pressure, precipitation, net radiation, wind speed, wind direction, and air temperature, relative humidity, air pressure, precipitation, net radiation, wind speed, and wind direction extracted from ERA5 (see Table 3). The isotope variables are not mass-weighted, except for Ivittuut and NEEM sites (Only daily data is available.). It is noted that ERA data is not available for Trinity Beach, Ivittuut and NEEM sites, since it is recorded in different time intervals.

Variables for measurements made on flight and ship transects are similar with those of stationary measurements. They are recorded in the following order: YYYYMMDDHHmm, Altitude (in cases of flight), Latitude, Longitude, water vapor mixing ratio, δ^18^O, standard deviation of δ^18^O, δD, standard deviation of δD, air temperature, relative humidity, air pressure, precipitation, net radiation, wind speed and wind direction. ERA5 was not provided for flight and ship transects. The meta file describes variable unit, measurement equipment, and additional description for each column.

### Website Interface

An interactive map enables the user to view individual site information and download the dataset. By clicking on the site of interest on the map, the user is directed to the digital data and the metafile for chosen site.

## Technical Validation

The vapour isotope analyzers used by the site investigators fall into two broad categories: tunable diode laser absorption spectroscopy (TDLAS) and cavity-enhanced near infrared laser absorption spectroscopy (CEILAS). Both techniques measure isotopologue concentrations on the principle of the Beer–Lambert Law of light absorption. Detailed information on the analyzer types is given in Table 4.

TDLAS analyzers were used in New Haven, Borden Beijing, Luancheng, Duolun, Great Mountain, Rosemount (TGA100A and TGA200, Campbell Scientific) and Las Palmas (ISOWAT II, self-made). Laboratory tests of the TGA analyzers show a 1-h precision (one standard deviation) of 1.1‰ for δD and 0.07‰ for δ^18^O at the dewpoint temperature of 15 °C^32^, and 0.2‰ for δ^18^O at the dewpoint temperature of −10.8 °C^19^.

The ISOWAT II tunable diode laser spectrometer was an advanced second-generation instrument based on the ISOWAT prototype, which was operated onboard the IAGOS-CARIBIC passenger aircraft^54^. ISOWAT II was used during the MUSICA aircraft campaign (Flight Cruise: Las Palmas). Instead of relying on laboratory bench-test uncertainty estimations which can significantly underestimate in-flight uncertainties, this instrument featured a custom-designed in-flight working-reference source to check the instrument performance several times throughout each flight and at different altitudes of the vertical profile. In addition, extensive pre- and post-flight calibrations were conducted on the ground using a custom-designed calibration gas source. Uncertainties of the in-flight data were dependent on absolute humidity, which changed by 2 orders of magnitude between 25000 ppm near surface and as low as 100 ppm in the upper troposphere. The humidity-dependent uncertainties are provided in the data archive for each 1-second data point. For the majority of in-flight measurements, we found the total uncertainty (combined relative and absolute uncertainties) to be around 10‰ for δD.

The Picarro analyzers use cavity-ring down spectroscopy (CRDS) and the LGR analyzers use a later CEILAS technique called off-axis integrated-cavity-output spectroscopy (OA-ICOS)^48^. LGR analyzers were used in Qianyanzhou, Taihu, Nagoya, Mapla and Ship-JARE site, while Picarro analyzers were used in Manitou Forests, Niwot Ridge, Kourovka, Rietholzbach, Camargue, Karlsruhe, Teide, Izana, Heihe, Gaoqiao, Mase, Taipei, Trinity Beach, Ivittuut, NEEM, Dome C, Ship-Bermuda, Ship-PIRATA, Ship-RARA, Ship-STRASSE, Ship-ACTIV and Flight-HyMeX SOP1. The key difference between these two types is that CRDS requires smaller cavity volumes due to its on-axis beam geometry while ICOS provides much faster time response and wider dynamical ranges. The precision for 10- min averages is better than 0.04‰ and 0.1‰ for δ^18^O and δD, respectively, for Picarro analyzers, while for LGR analyzers, the precisions on δ^18^O and δD measurements are about 0.02‰ and 0.20‰, respectively^48^, although this hugely depends on the humidity level and there needs to be frequent calibration to reach this point.

Four of the stationary sites have documented cross-validation against other measurement techniques. (1) In Beijing, the difference between the laser measurement and cold trap/mass-spectrometer analysis is 1.2 ± 6.0‰ for δD and 1.1 ± 1.3‰ for δ^18^O, and the difference between the laser measurement and that predicted from a Rayleigh distillation device is 1.1‰ for D/H and −0.06‰ for ^18^O/^16^O^32^. (2) In New Haven, a comparison of the laser measurement of δ^18^O with cold trap/mass-spectrometry and a Rayleigh distillation device reveals a difference of −0.36 and −0.11‰, respectively^55^. (3) At Dome C, Allan-Werle variance measurements of the Picarro L2130i analyzer and a high-finesse water isotope spectrometer (HiFI; based on the technique of optical feedback cavity-enhanced absorption spectroscopy developed in Laboratoire Interdisciplinaire de Physique, Grenoble, France) shows almost no loss of precision with the analyzer down to a humidity of 450 ppmv (0.2‰ for δ^18^O and 1.1‰ for δD) to 200 ppmv. (4) At the NEEM site, inter-comparison between Picarro L1102-I and LGR 100 A analyzers (with every 1.5 h calibration) reveals acceptable reproducibility, stability, and precision with standard deviations of 0.23‰ for δ^18^O and 1.4‰ for δD.

While measurement errors can be related to fundamental optical effects such as the coherent nature of laser radiation, the main errors in the isotopic field measurements arise from two sources: (1) absolute isotope correction or standardization to the VSMOW scale, and (2) humidity dependence. Calibration procedures for removing these errors vary between instruments as well as over time. Humidity-dependent calibrations were not always applied, but all measurements were calibrated and reported to the VSMOW scale. The calibration cycle is typically 3 to 6 h, by introducing one or more reference waters into a vapourizer.

**Figure 3:**
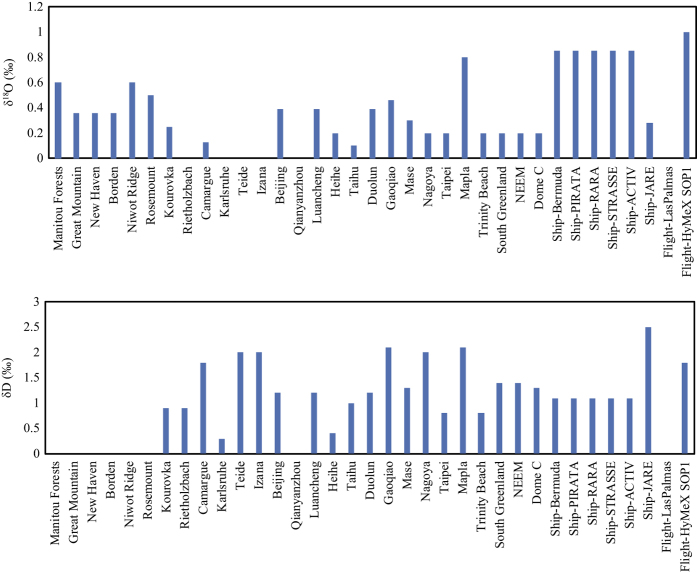
Uncertainties of isotopic data reported in this dataset, Flight – Las Palmas uncertainties were humidity dependent.

In CEILAS-based analysis, multiple isotope standards are used for calibration. During the calibration cycle, the analyzer is switched from the ambient vapour sample to the calibration reference stream. Correction to the absolute VSMOW scale and removal of instrument drift are made with a curve fit between the measured values and the known values of the water standards. At a number of sites (Manitou Forests, Kourovka, Rietholzbach, Niwot Ridge, Camargue, Teide, Izana, Gaoqiao, Mase, Ivittuut, Ship-PIRATA, Ship-STRASSE), the calibration was accomplished with an automated Picarro Standards Delivery Module (SDM). At Nagoya, Mapla, Trinity Beach, NEEM, Ship-JARE and Flight-HyMeX SOP1, an LGR water vapour isotope standard source (DLT100, LGR) with a single or multiple water standards was used for calibration. Some sites, including Borden, New Haven, Great Mountain, Rosemount, Beijing, Luancheng, Ship-Bermuda, Ship-RARA, Ship-ACTIV and Flight-LasPalmas, used a self-made calibration module. Due to difficulties in field operation, the analyzer at Dome C was calibrated in the laboratory after field deployment. The delta values of the calibration standard waters used in each site are summarized in Table 4. As shown in Table 4, some sites used multiple water standards to correct for instrumental drift by altering the VSMOW-scaling offset, while a few other sites used a single water standard, effectively treating “drift” as white noise that adds to measurement uncertainty. The mean uncertainties given in Fig. 3 indicate the overall accuracy of the isotopic measurement during the study period for each site. These uncertainty values include errors propagated through the calibration procedure. It is noted that since all datasets are robustly quality controlled and calibrated typically 3 to 6 h interval, these uncertainties are just given as a reference.

Humidity dependence is characterized by measuring a reference water vapour stream of the same isotopic compositions across a range of humidity conditions^27,48,51^. The level of humidity dependence is instrument-specific. Generally, humidity dependence is less significant at humidity levels greater than 5000 ppmv. For drier situations where humidity varies between 2000–5000 ppmv, errors on the order of 1.8‰ for D and 0.6‰ for ^18^O can be expected^56^. At very low humidity levels (below 2000 ppmv), standard calibration devices, such as the SDM from Picarro, are not able to generate stable humidity, and confidence is low for composite isotopic variables such as deuterium excess and custom-made calibration devices has therefore been developed.

In Manitou Forests, Niwot Ridge, Kourovka, Rietholzbach, Camargue, Karlsruhe, Nagoya, Mapla, NEEM, Ivittuut, Dome C, Kohnen, ship cruises (JARE, STRASSE, PIRATA, RARA, ACTIV and Bermuda) and flight cruises (LasPalmas and HyMeX SOP1), corrections were made for humidity dependence, assuming that the measurement drift is linear or non-linear function of water vapour concentration. Another procedure for removing the humidity dependence is to dynamically adjust the humidity of the calibration stream to match the humidity of the ambient air. This dynamic calibration system is referred to as the ‘dripper’ by Lee *et al*.^19^. This system was used in Beijing, Duolun, Heihe, Luancheng, Great Mountain, New Haven, Qianyanzhou and Rosemount. For Teide, Izana, Mase, Gaoqiao, TrinityBeach and Taipei sites, no humidity correction was applied. The humidity dependence correction information also available in Meta file for each site.

## Usage Notes

The datasets archived on this site are contributed by individual scientists and institutes and have been quality-controlled by the site investigators. In most cases, the data have been published in peer-reviewed journals. We encourage interested parties to contact the site investigators to explore possible collaboration opportunities based on these data.

## Additional Information

**How to cite this article**: Wei, Z. *et al*. A global database of water vapor isotopes measured with high temporal resolution infrared laser spectroscopy. *Sci. Data*. 6:180302 https://doi.org/10.1038/sdata.2018.302 (2019).

**Publisher’s note**: Springer Nature remains neutral with regard to jurisdictional claims in published maps and institutional affiliations.

## Supplementary Material



## Figures and Tables

**Table 1:** List of datasets as of March 2018.

Site Name	Continent/Cruises	Location	Lat	Lon	Landscape	Climate
Manitou Forests	North America	Colorado, USA	39.08	−105.07	Forest	Bsk
Great Mountain	North America	Connecticut, USA	41.98	−73.23	Forest	Dfb
New Haven	North America	Connecticut, USA	41.31	−72.92	Urban	Dfa
Borden	North America	Borden, Canada	44.32	−79.93	Forest	Dfb
Niwot Ridge	North America	Colorado, USA	40.03	−105.55	Forest	Dfc
Rosemount	North America	MN, USA	44.72	−93.10	Cropland	Dfa
Kourovka	Europe	Kourovka, Russia	57.04	59.55	Forest	Dfb
Rietholzbach	Europe	Swiss Prealps, Swiss	47.38	8.99	Grassland	ET
Camargue	Europe	Vaccarès Lagoon, France	43.60	4.47	Wetland	Csa
Karlsruhe	Europe	Karlsruhe, Germany	49.10	8.44	Snow	Cfb
Teide	Europe	Canary Islands, Spain	28.3	−16.5	Island	BWk
Izana	Europe	Canary Islands, Spain	28.3	−16.5	Island	BWk
Beijing	Asia	Beijing, China	40.00	116.38	Urban	Dwa
Qianyanzhou	Asia	Jiangxi, China	26.75	115.06	Forest	Cfa
Luancheng	Asia	Shijiazhuang, China	37.88	114.68	Cropland	Bsk
Heihe	Asia	Hehei, China	38.85	100.37	Cropland	Bwk
Taihu	Asia	Jiangsu, China	30.09	119.88	Lake	Cfa
Duolun	Asia	Inner Mongolia, China	42.03	116.28	Grassland	Dwb
Gaoqiao	Asia	Zhejiang, China	21.57	109.76	Wetland	Cfa
Mase	Asia	Tsukuba, Japan	36.05	140.03	Cropland	Cfa
Nagoya	Asia	Nagoya, Japan	35.15	136.97	Urban	Cfa
Taipei	Asia	Taipei, ROC	25.04	121.74	Coast	Cfa
Mapla	Africa	Mapla, Kenya	0.4	36.87	Savanna	AW
Trinity Beach	Oceania	Cairns, Australia	−16.79	145.70	Coast	AM
NEEM	North pole	Greenland	77.45	−51.05	Ice Sheet	ETf
Ivittuut	North pole	Greenland	61.20	−47.18	Coast	ET
Dome C	Antarctic Pole	Concordia, Antarctica	−75.10	123.38	Ice Sheet	ETf
Kohnen	Antarctic Pole	Antarctica continent	−70.0	0.0	Ice Sheet	ETf
Ship-Bermuda	Flight and ship cruises	Atlantic	32.05	−64.43	Ocean	–
Ship-PIRATAFR24	Flight and ship cruises	Atlantic	5.07	−4.00	Ocean	–
Ship-RARA AVIS	Flight and ship cruises	Atlantic	48.37	−4.49	Ocean	–
Ship-STRASSE	Flight and ship cruises	Atlantic	27.51	−17.10	Ocean	–
Ship-ACTIV	Flight and ship cruises	Atlantic	56.18	12.33	Ocean	–
Ship-JARE	Flight and ship cruises	Australia and Syowa base	−31.97	115.68	Ocean	–
Flight-Las Palmas	Flight and ship cruises	Canary Islands, Spain	27.9	−15.37	Flight	–
Flight-HyMeX SOP1	Flight and ship cruises	Corsica, France	42.55	9.48	Flight	Csa
The measurement sites are grouped by continent and in relation to the Köppen climate zones: AW: Tropical wet and dry or savanna climate; AM: Tropical monsoon climate; BWh: Hot desert climate; BWk: Cold desert climate; BSh: Hot semi-arid climate; BSk: Cold semi-arid climate; Cfa: Humid subtropical climate; Csb: Warm-summer Mediterranean climate; Dwa: Monsoon-influenced hot-summer humid continental climate; Dwb: Monsoon-influenced warm-summer humid continental climate; Dfa: Hot-summer humid continental climate; Dfb: Warm-summer humid continental climate; Dfc: Subarctic climate; ET: Polar Tundra; ETf: Polar/frost.						

**Table 2:** List of meteorological measurements and sensor types.

Site Name	Continent/Cruises	Air temperature	Relative humidity	Pressure	precipitation	Net radiation	Wind speed, Wind direction
Great Mountain	North America	HMP-45C			Tipping bucket	Q7	RMY sensor
New Haven	North America	Data from Tweed airport, New Haven					
Niwot Ridge	North America	HMP-35D		PTB-101B	USCRN Data	REBS Q-7.1	CSAT3
Manitou Forests	North America	HMP-35D		PTB-101B	Tipping bucket	REBS Q-7.1	CSAT3
Rosemount	North America	HMP - 45 C			TE525MM	CNR-1	DA600-62AX
Borden	North America	HMP - 45 A			Tipping bucket	CNR-1	CSAT3
Camargue	Europe	HRT2122 (Précis-Mécanique)		–	3029/2 (Précis-Mécanique)	–	Meteo-France station
Rietholzbach	Europe	–					
Kourovka	Europe	MetPak-II					
Karlsruhe	Europe	Mirror hygrometer	HMP-45A	From IMK-TRO			
Teide	Europe	Rotronic	Rotronic	Setra 470	–	Kipp & Zonen CM-5 and CM-11	Thies Sonic anemometer
Izana	Europe	Rotronic	Rotronic	Setra 470	–	Kipp & Zonen CM-5 and CM-11	Thies Sonic anemometer
Beijing	Asia	Davis weather station			–	–	
Qianyanzhou	Asia	HMP-45C			–	–	–
Luancheng	Asia	HMP-45C			TE525MM	CNR-1	A100R
Duolun	Asia	HMP-45C		CS105	TE525MM	CNR-1	A100R
Heihe	Asia	–					
Taihu	Asia	HMP-45C		–	TE525MM	CNR-1	CSAT3
Gaoqiao	Asia	HMP-45A			TE525MM	CNR-1	010 C Met One
Mase	Asia	HMP-45A			TE525MM	CNR-1	DA600-62AX
Nagoya	Asia	–					
Taipei	Asia	CWB Taipei (station code: 466920)					
Mapla	Africa	–					
Trinity Beach	Oceania	HMP45C			Tipping bucket	CNR4	CSAT3
NEEM	North pole	–					
Ivittuut	North pole	–					
Dome C	Antarctic Pole	HMP155	–				
Kohnen	Antarctic Pole	–					
ACTIV	Flight and ship cruises	Davis			–	–	–
Ship-Bermuda	Flight and ship cruises	RM Young weather station					–
Ship-PIRATAFR24	Flight and ship cruises	BATOS (French Met Office) weather station					
Ship-RARA AVIS	Flight and ship cruises	MetPak Pro weather station from Gill Instruments					
Ship-STRASSE	Flight and ship cruises	BATOS (French Met Office) weather station					
Ship-JARE	Flight and ship cruises	Batos weather station					
Flight-Las Palmas	Flight and ship cruises	–	KT15	–	–	CNR-1	–
Flight-HyMeX SOP1	Flight and ship cruises	PT100	HMP 233	Five-hole probe	–	–	Flight

**Table 3:** List of variable names and measurement details for the Mase site.

Column	Description	Unit	Equipment	Height (m)	Additional description
Column 1	Start time	−	−	−	UTC
Column 2	End time	−	−	−	UTC
Column 3	Water vapour isotopic ratio (18 O)	per mil	Picarro L2120-i	2	Normalized to V-SMOW; No humidity dependent correction applied.
Column 4	Standard deviation of 18 O	per mil	Picarro L2120-i	2	Hourly
Column 5	Water vapour isotopic ratio (D)	per mil	Picarro L2120-i	2	Normalized to V-SMOW; No humidity dependent correction applied
Column 6	Standard deviation of D	per mil	Picarro L2120-i	2	Hourly
Column 7	Air temperature	Celsius (°C)	HMP-45A	3.9	−
Column 8	Relative humidity	<= 1	HMP45A	3.9	−
Column 9	Air pressure	kPa	HMP45A	3.9	−
Column 10	Precipitation	mm	TE525MM	−	−
Column 11	Net radiation	W/m^2^	CNR-1	2.35	−
Column 12	Wind speed	m/s	DA600-62AX	3.9	−
Column 13	Wind direction	°C	DA600-62AX	4.9	−
Column 14	Air temperature	Celsius (°C)	–	2	ERA5
Column 15	Relative humidity	<= 1	–	2	ERA5
Column 16	Air pressure	kPa	–	–	ERA5
Column 17	Precipitation	mm	–	−	ERA5
Column 18	Net radiation	W/m^2^	–	–	ERA5
Column 19	Wind speed	m/s	–	10	ERA5
Column 20	Wind direction	°C	–	10	ERA5

**Table 4:** List of isotopic analyser type, calibration module and isotopic compositions of standard calibration water.

Site	Continent/Cruises	Analyzer	Calibration Module	Standards (‰)		Uncertainty	
				δ^18^O	δD	δ^18^O	δD
Manitou Forests	North America	Picarro L2120i	PSCM	−13.1; −49.7	−97.0; −388.0	0.6	−
Great Mountain	North America	Campbell TGA100A	DCS	−6.66	−	0.36	−
New Haven	North America	Campbell TGA100A	DCS	−15.8	−	0.36	−
Borden	North America	Campbell TGA100A	Self-made	−1.037; −11.861	−	0.36	−
Niwot Ridge	North America	Picarro L2120i	PSCM	−13.1; −49.7	−97.0; −388.0	0.6	−
Rosemount	North America	Campbell TGA100A and TGA200	DCS	−8.5	−60.0	0.5	−
Kourovka	Europe	Picarro L2130i	PSCM	−12.76; −36.71; −54.05	−96.4; −289.0; −424.1	0.25	0.9
Rietholzbach	Europe	Picarro L1115i	PSCM	−10.99; −24.89; −46.02	−78.68; −153.90; −256.11	0. 23	0.9
Camargue	Europe	Picarro L1102i	Self-made	−17.12; −7.85; −0.68	−133.3; −53.5; 3.7	0.13	1.8
Karlsruhe	Europe	Picarro L2120-i	PSCM	−9.09; −18.90; −33.43	−62.1; −142.2;−245.7	−	0.3
Teide	Europe	Picarro L2120-i	PSCM	−18.90; −33.43	−142.2; −245.7	−	2
Izana	Europe	Picarro L2120-i	PSCM	−18.90; −33.43	−142.2; −245.7	−	2
Beijing	Asia	Campbell TGA100A	DCS	−14.13	−104.0	0.39	1.2
Qianyanzhou	Asia	LGR DLT100	DCS	–13.55	–101.7	−	−
Luancheng	Asia	Campbell TGA100A	DCS	−14.13	−104.0	0.39	1.2
Heihe	Asia	Picarro L1102i	DCS	−	−	0.2	0.4
Taihu	Asia	Campbell TGA100A	DCS	−32.99; −6.12	−256.0; −43.2	0.1	1
Duolun	Asia	Campbell TGA100A	DCS	−14.13	−104.0	0.39	1.2
Gaoqiao	Asia	Picarro L2130i	PSCM	−7.07; −4.29	−49.68; −19.46	0.1	1
Mase	Asia	Picarro L2120i	PSCM	−0.3; −26.0	−1.2; −197.9	0.3	1.3
Nagoya	Asia	LGR DLT100	WVISS	−11.3	−78.0	0.2	2
Taipei	Asia	Picarro L2120i	Self-made	−7.8; −12.0; −14.1; −16.0; −19.2	−59.0; −85.4; −102.3; −119.2; −145.1	0.2	0.8
Mapla	Africa	LGR DLT100	WVISS	−19.57; −2.96	−154.1; −9.8	0.8	2.1
Trinity Beach	Oceania	Picarro L2130i	WVISS	+ 0.88; −10.64; −18.36	+ 3.7; −71.5; −140.4	0.2	0.8
Ivittuut	North pole	Picarro L2120i	PSCM	−6.12; −32.99	−43.2; −256.0	0.2	1.4
NEEM	North pole	Picarro L1102i	WVISS	−6.12	−43.2	0.2	1.4
Dome C	Antarctic Pole	Picarro L2130i	Self-made + Cryogenic trapping comparison	−54.30; −33.56	−431.1; −257.6	0.2^∗^	1.3^∗^
Kohnen	Antarctic Pole	LGR DTL-100	WVISS	−44.44	−345.5	0.9	3.0
Ship-Bermuda	Flight and ship cruises	Picarro L2120i	Self-made	+ 0.40; −33.56; −54.05	+ 2.8; −257.6; −424.1	0.85	1.1
Ship-PIRATA	Flight and ship cruises	Picarro L2130i	PSCM	−3.26; −6.61; −14.05	−21.32; −44.32; −100.96	0.85	1.1
Ship-RARA	Flight and ship cruises	Picarro L2120i	Self-made	−6.61; −14.05; −18.71; −19.74	−44.32; −100.96; −144.7; −146.53	0.85	1.1
Ship-STRASSE	Flight and ship cruises	Picarro L2130i	PSCM	−0.56; −6.61; −15.81	−3.75; −44.32; −120.68	0.85	1.1
Ship-ACTIV	Flight and ship cruises	Picarro L1102i	Self-made	+ 0.40; −0.02; −14.84; −33.44; −54.05	+ 2.8; −65.0 ; −111.1; −257.3 ; −424.1	0.85	1.1
Ship-JARE	Flight and ship cruises	LGR DLT100	WVISS	−11.3	−78.0	0.28	2.5
Flight-LasPalmas	Flight and ship cruises	ISOWAT	Self-made	−	−59.22	–	10^∗^
Flight-HyMeX SOP1	Flight and ship cruises	Picarro L2130i	WVISS	−11.0; −70.2	−78.7; −166.7	1	1.8
WVISS: LGR water vapour isotope standard source; DCS: dripper calibration system; PSCM: Picarro standard calibration module. ^∗^Laboratory tests.							
^∗∗^Uncertainty was humidity dependent. Most data were certain to within 10‰ for δD (combined relative and absolute uncertainties).							
